# Developing a veterinary professional “identity”: first year veterinary students’ perspectives on how they describe themselves now and as future veterinarians: 2017–2024

**DOI:** 10.3389/fvets.2025.1614449

**Published:** 2025-09-16

**Authors:** Rodney S. Bagley, Joyce Carnevale, Amelia Mindthoff

**Affiliations:** ^1^Department of Veterinary Clinical Sciences, College of Veterinary Medicine at Iowa State University, Ames, IA, United States; ^2^Office of Academic and Student Affairs, College of Veterinary Medicine at Iowa State University, Ames, IA, United States

**Keywords:** veterinary student perspectives, descriptive words, professional identity, self-reflection, word choices

## Abstract

**Introduction:**

As an assignment in an introductory course to the profession, we asked first year veterinary students in their initial (fall) semester to identify words that they would use to describe themselves now and how they want to be described in the future as veterinarians.

**Methods:**

Using a Qualtrics survey instrument, students were asked to record five descriptive words in response to each of the following prompts: “How would you describe yourself?”, “How would you like your future veterinary colleagues to describe you?”, and “How would you like your future clients to describe you?” Students’ responses were collected beginning in fall of 2017 (Class of 2021) through fall of 2024 (Class of 2028). Word choices were collated and ranked based on the number of times an individual word was recorded.

**Results:**

While there was some variability from year to year, the five most common word choices for the prompt “How would you describe yourself?” were: “Hard-working”, “Kind”, “Caring”, “Compassionate”, and “Determined”. The five most common word choices for the prompt “How would you like your future veterinary colleagues to describe you?” were: “Hard-working”, “Compassionate”, “Knowledgeable”, “Caring”, and “Reliable”. The five most common word choices for the prompt “How would you like your future clients to describe you?” were: “Compassionate,” “Knowledgeable”, “Caring”, “Kind”, and “Empathetic”. The recorded rank for the most common descriptive word changed slightly over the study time to the prompts “how would you describe yourself” and the “how would you want your colleagues to describe you”, but not the “how would you want your clients to describe you” prompt.

**Discussion:**

These collective student responses provide insight into the origin of their veterinary “identity” as they enter the veterinary curriculum to become veterinarian professionals.

## Introduction

How veterinary students become practicing veterinary professionals is a multifaceted, dynamic, and complex process involving the development of both technical and non-technical skills. In many ways, however, the process remains enigmatic. In addition to the classic skill development process, there is a transition in thinking about the profession and how one identifies within the profession. A component of this development is the formation of a professional “self,” “brand,” or a professional “identity” (2, 4, 17, 44, 48, 49, 62). As a professional “identity” does not always have one standard definition; this concept is often articulated in broad constructs based on what professionals do (actions and behaviors); the possession of certain knowledge and skills; a set of values, beliefs, and ethics; a social identity; and a group identity (17). A professional identity has elements of a professional “self-concept” and is a process of identification of “self” within the profession. A professional identity is formed as an individual internalizes the characteristics, norms, and values of the profession (12). This involves a process of students defining who they are in the profession and occurs in established communities such as universities and hospitals (19). This sense of belonging as a professional can lead to important outcomes such as job satisfaction, improved patient safety, decreased stress, and improved retention and recruitment of practitioners (11, 48, 63, 66). In the educational setting, developing a professional identity may result in students trying harder, increasing student success, and increased student retention (11, 63, 66). A professional identity can be formed through numerous explicit and implicit experiences. Therefore, every experience a veterinary student has with a veterinarian or the veterinary profession influences this identity. Developing professionals evolve their professional identity through education and experiences, which influence who they become as veterinary professionals. This includes important aspects of the actions, behaviors, and communication dynamics between themselves and others, such as colleagues and clients.

How one aspires to be described as a professional may serve as a motivation for individual skill development and influence future employability (1, 7, 22, 26, 39) and may also influence similar aligned constructs, such as professional and personal “branding” (5, 20). Unique words one chooses to describe themselves concurrently provide others an opportunity to gather insight about the individual’s thought process, self-awareness, self-concept, and aspirational personal goals (15, 21, 24, 27, 34, 43, 64).

In this study, we examined a stage in the development of the veterinary professional “identity” at the early formal educational process by engaging first-year veterinary students to describe themselves now and how they envision themselves being described as future veterinarians. We provided this educational opportunity to support developing their views on their professional “identity.” Our goal in codifying this information was to improve our understanding of how students at the beginning of their veterinary training view themselves prior to subsequent influences provided in the curriculum and allied veterinary career experiences.

To gain insight into our students related to their professional “identity,” we asked first-year veterinary students to provide self-descriptive word choices for themselves today and as a future veterinarian. We asked first-semester veterinary students to choose single words to describe themselves now and how they would like to be described as future veterinarians from both a colleague’s and a client’s perspective. For the assignment, veterinary students were asked to record five single descriptive word choices based on three prompts:

“How would you describe yourself?”

“How would you like your future veterinary colleagues to describe you?”

“How would you like your future clients to describe you?”

We were most interested in empirically determining (1) the unique word choices students use to describe themselves personally at the beginning of their veterinary education, (2) how they envision being described professionally in the future, and (3) if the most common descriptors were preserved or evolved over the study time. The hypothesis for comparison is that the most common student word choices would not change over the study time, even as new generations of students enter the veterinary curriculum.

## Materials and methods

This study was submitted for the Iowa State University Human Subjects Institutional Review Board (IRB 23–381) review and deemed exempt. In collecting this data, first-year veterinary students at Iowa State University College of Veterinary Medicine were asked to record five descriptive words to three posed questions. For each cohort (year), student responses were collected during the fall of their first year during the month of September, which was approximately, depending on the year, the third to fourth week of their veterinary curriculum. As such, the year presented in analyses refers to the respective cohort’s initial year in the veterinary curriculum and the year that the data was obtained. The data were collected as a course assignment during a core introductory course to the veterinary career. This course is colloquially entitled “Careers and Career Success,” in which students are introduced to various aspects of veterinary career options and are provided self-reflective assignments relative to aspects of the veterinary profession and their future success therein. Pilot data were collected from first-year students entering the curriculum in 2016 to refine the final study questions and determine compliance in completing the assignment.

Prior to presenting the questions posed, we provided no explicit word choice examples so that students had individual intellectual freedom to choose descriptive words in an unbiased manner. Questions were open-ended, allowing for unstructured “stream of consciousness” or “natural” answers relative to the word choices recorded. Words were recorded into a text entry box format. The students were instructed that there was no “right/wrong or correct/incorrect” answer to the questions posed to allow for spontaneous responses. Class credit for the assignment was earned by completion of the assignment only and not based on the content of the answer. Students were provided seven calendar days once the assignment was available to complete the assignment.

For the assignment, students were provided with a Qualtrics^1^ survey instrument and asked to record five single descriptive words in a text entry box for each of the following prompts:

**Question 1.** “How would you describe yourself?”

**Question 2.** “How would you like your future veterinary colleagues to describe you?”

**Question 3.** “How would you like your future clients to describe you?”

The questions were asked sequentially in the order shown here. The students were asked these same three questions in the same format annually from the fall of 2017 through the fall of 2024.

## Analysis

Considering the subjective nature of the responses and that these data were from a unique single US institution, we employed descriptive statistical methodologies for this evaluation. These encompassed quantitative measures, such as counts, averages, and percentages. We did not match individually identifiable information with the responses, except that the student’s name was recorded with the assignment to provide them with credit for completion of the assignment. For this analysis, the student’s name was subsequently removed from the data set prior to collective aggregation. For each year and each of the questions, all descriptive words were grouped together into one cohort data set, as we did not ask the students to rank or prioritize the relative rank of any individual descriptor compared with the others. From each grouped dataset, we sub-grouped the same word or word stem together and then counted the absolute number of instances that an individual word was recorded by the student cohort/year. When the equivalent noun was used instead of the adjective (i.e., “compassion” vs. “compassionate”), we converted the noun form to the adjective form for counting. The sum of the unique individual words recorded was divided by the number of students who completed the assignment to determine the percentage of the total students who recorded that individual unique word per year as one of their five descriptors. Finally, the 10 most common word choices (based on the number of occurrences of a unique individual word in the complete dataset) were ranked for each cohort year from the most frequently recorded to the least frequently recorded ones.

## Results

As this was a required class assignment, there was no dropout rate, as all students in each class completed the survey. The number of students who completed the assignment varied per year based on class size (between 130 and 151 per year). In total, there were 1,098 individual student responses for this analysis.

### Student general demographics

**Figure 1:**
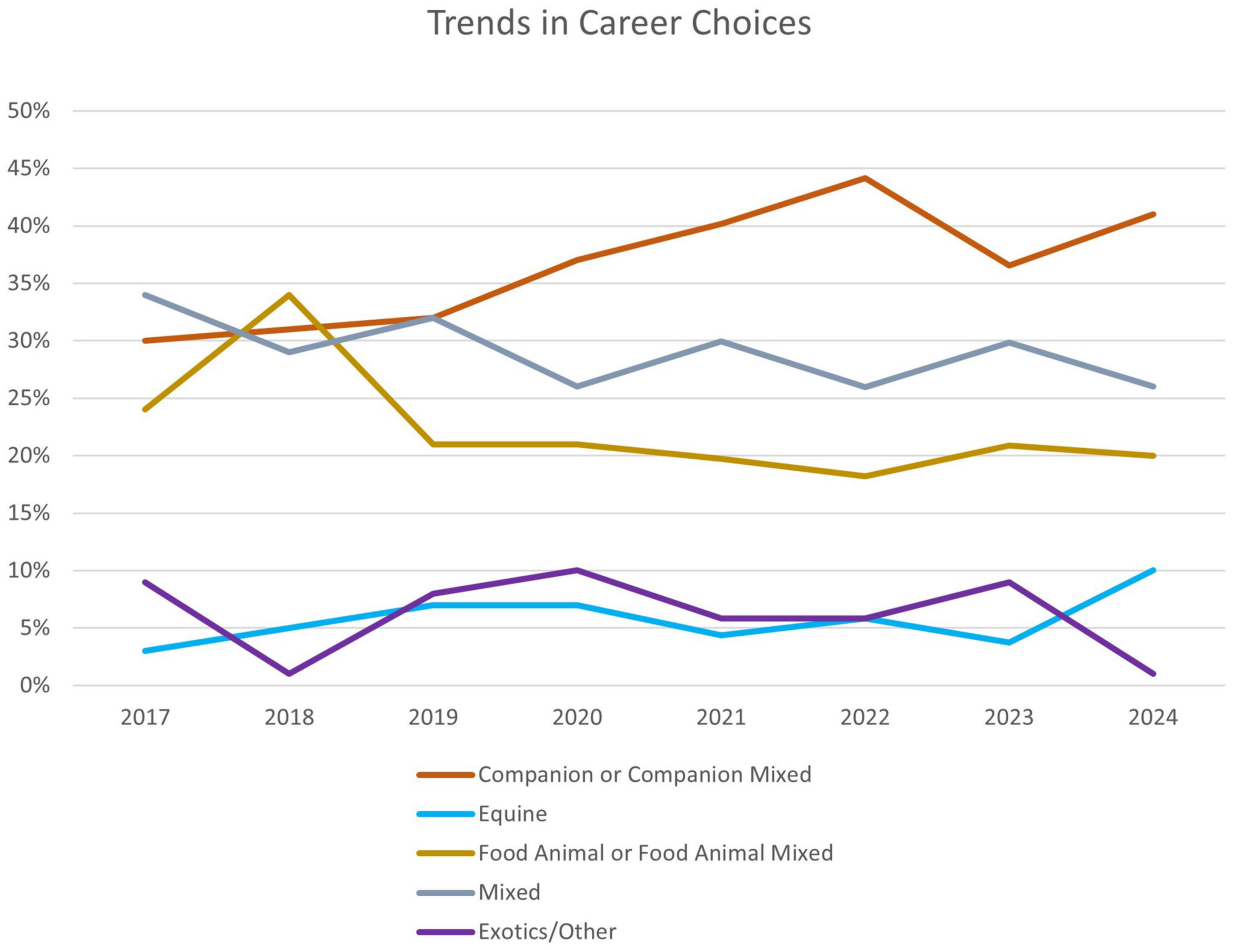
Trends in anticipated career choices.

We did not match individual student demographics or other individually identifiable information with the responses, as the student’s name was removed from the dataset prior to analysis. We did not, therefore, correlate any unique identifiable student information and any individual response. For general context relative to the characteristics of each cohort, we present the age and gender distributions for each of the classes within our sample, as self-reported in admissions data collected at the time of matriculation into the first semester of the veterinary curriculum (Table 1). Gender distribution did not greatly vary across the years, with most students in each of the classes identifying as female (81.7–85.8%, depending on the class year). The average age of students was consistent across the classes as well (23.5–24.4 years of age). Given that we did not identify individual answers by gender and age across the classes, we did not include these variables as covariates within our analyses. In a separate class assignment, students were asked about their planned practice species (Table 2; Figure 1). This information was similarly not directly linked to the word choice data in this study. Therefore, we did not correlative species of interest and word choices recorded and provide this here for the general context of the population of students.

**Table 1:** Demographic information from each cohort.

Year	Gender		Age	
	Men	Women	Average	Range
2017	14.8%	85.2%	24.0	21–47
2018	14.1%	85.2%	23.8	20–53
2019	16.8%	83.2%	23.9	20–40
2020	17.7%	82.3%	24.4	20–46
2021	18.3%	81.7%	23.5	20–35
2022	17.3%	82.7%	24.2	20–44
2023	14.2%	85.8%	24.3	20–49
2024	17.3%	82.7%	24.1	21–42

Self-identified data. For both 2018 and 2024, one student declined reporting their gender.

**Table 2:** Percentages of students anticipated career choices.

Students anticipated career choice	2017	2018	2019	2020	2021	2022	2023	2024
Companion or companion mixed	30%	31%	32%	37%	40%	44%	37%	41%
Equine	3%	5%	7%	7%	4%	6%	4%	10%
Food animal or food animal mixed	24%	34%	21%	21%	20%	18%	21%	20%
Mixed	34%	29%	32%	26%	30%	26%	30%	26%
Exotics/other	9%	1%	8%	10%	6%	6%	9%	1%

### Unique word choices

Students in each class were recorded between 139 and up to 210 unique words as descriptors for each of the three prompts (Table 3). No student recorded the same word more than once in any of their responses. For all years combined, students chose an average of 182, 185, and 156 unique words with a median of 184, 184, and 156 unique words to answer each of three questions (Question one, Question two, and Question three, respectively). For the questions “How would you describe yourself?” (Question one) and “How would you like your future veterinary colleagues to describe you?” (Question two), students recorded approximately the same number of unique words (median of 184), whereas for the prompt “How would you like your future clients to describe you?” (Question three), students recorded fewer unique words (with a median of 156 words—approximately 15% less unique word choices compared with the other questions responses).

**Table 3:** Number of unique word choices for each question/year.

Year	2017	2018	2019	2020	2021	2022	2023	2024		
Prompt									Total mean	Total median
How do I describe myself?	166	175	166	191	192	191	176	197	182	184
How will my colleagues in 5 years describe me?	210	175	184	183	194	190	167	174	185	184
How will my clients in 5 years describe me?	166	155	161	149	145	174	139	156	156	156

The top 10 word choices based on absolute counts relative to how many students recorded that unique word for each of the prompts/student cohort are shown in Tables 4–6. Combining all years, the 10 most common words used to describe themselves based on the prompt “How would you describe yourself?,” beginning with most commonly recorded, were “Hard-working,” “Kind,” “Caring,” “Compassionate,” “Determined,” “Dedicated,” “Passionate,” “Empathetic,” “Helpful,” and “Reliable.” The most common 10 words used to describe themselves based on the prompt “How would you like your future veterinary colleagues to describe you?,” beginning with most recorded, were “Hard-working,” “Compassionate,” “Knowledgeable,” “Caring,” “Reliable,” “Passionate,” “Dedicated,” “Helpful,” “Intelligent,” and “Kind.” The most common 10 words used to describe themselves based on the prompt ““How would you like your future clients to describe you?,” beginning with most recorded, were “Compassionate,” “Knowledgeable,” “Caring,” “Kind,” “Empathetic” “Helpful,” “Intelligent,” “Trustworthy,” “Friendly,” and “Honest.” Following the initial analysis, the number of responses for the word “Compassionate” was combined with number of responses for the word “Empathetic” as these word choices are often used interchangeably by individuals to describe the same “caring” concept. The combination of these word choices was the most recorded overall word choice in all years in the two future-self questions (i.e., envisioned descriptions by colleagues and clients) over this study period (Table 7).

**Table 4:** Most common word choices (ranked) for the prompt “How would you describe yourself?” following each word, the percentage of students using that word is in parentheses.

Year	2017	2018	2019	2020	2021	2022	2023	2024
Number of students	130	132	141	137	141	151	131	135
Rank								
1	Hard-working (43%)	Hard-working (50%)	Hard-working (43%)	Hard-working (31%)	Compassionate (26%)	Hard-working (22%)	Kind (21%)	Hard-working (23%)
2	Determined (15%)	Helpful (18%)	Dedicated (21%)	Passionate (22%)	Caring (21%)	Caring (20%)	Compassionate (18%)	Kind (23%)
3	Responsible (15%)	Dedicated (15%)	Reliable (21%)	Caring (20%)	Kind (19%)	Kind (1%)	Hard-working (18%)	Determined (21%)
4	Dedicated (12%)	Friendly (14%)	Kind (13%)	Compassionate (19%)	Hard-working (17%)	Compassionate (1%)	Empathetic (18%)	Caring (18%)
5	Reliable (12%)	Kind (13%)	Caring (12%)	Kind (18%)	Passionate (16%)	Passionate (1%)	Determined (17%)	Compassionate (17%)
6	Helpful (12%)	Reliable (12%)	Helpful (12%)	Dedicated (15%)	Determined (16%)	Determined (1%)	Intelligent (17%)	Empathetic (16%)
7	Friendly (11%)	Caring (11%)	Positive (11%)	Determined (14%)	Empathetic (12%)	Empathetic (1%)	Passionate (17%)	Passionate (15%)
8	Motivated (11%)	Compassionate (11%)	Happy (11%)	Intelligent (13%)	Dedicated (11%)	Honest (1%)	Caring (15%)	Loyal (13%)
9	Dependable (10%)	Determined (11%)	Passionate (11%)	Driven (12%)	Driven (11%)	Driven (1%)	Ambitious (11%)	Driven (12%)
10	Professional (9%)	Motivated (10%)	Compassionate (10%)	Empathetic (12%)	Trustworthy (10%)	Intelligent (1%)	Driven (11%)	Organized (12%)

**Table 5:** Most common word choices for the prompt “How would you like your future veterinary colleagues to describe you?” following each word, the percentage of students using that word is in parentheses.

Year	2017	2018	2019	2020	2021	2022	2023	2024
Number of students	130	132	141	137	141	151	131	135
Rank								
1	Knowledgeable (27%)	Hard-working (32%)	Knowledgeable (28%)	Hardworking (30%)	Compassionate (29%)	Compassionate (23%)	Compassionate (21%)	Compassionate (24%)
2	Hardworking (22%)	Knowledgeable (28%)	Hardworking (24%)	Knowledgeable (21%)	Knowledgeable (24%)	Hard-working (19%)	Hard-working (18%)	Hard-working (23%)
3	Compassionate (18%)	Compassionate (19%)	Dedicated (19%)	Compassionate (19%)	Hard-working (23%)	Caring (18%)	Passionate (16%)	Dedicated (20%)
4	Intelligent (18%)	Dedicated (16%)	Compassionate (18%)	Reliable (18%)	Passionate (23%)	Knowledgeable (17%)	Intelligent (14%)	Reliable (20%)
5	Caring (14%)	Helpful (15%)	Reliable (16%)	Caring (16%)	Helpful (13%)	Passionate (16%)	Caring (14%)	Caring (19%)
6	Passionate (12%)	Passionate (13%)	Helpful (16%)	Dedicated (15%)	Caring (12%)	Dependable (13%)	Knowledgeable (13%)	Kind (17%)
7	Trustworthy (12%)	Reliable (13%)	Kind (15%)	Intelligent (15%)	Dedicated (12%)	Helpful (11%)	Kind (12%)	Knowledgeable (13%)
8	Helpful (12%)	Intelligent (12%)	Empathetic (13%)	Passionate (15%)	Reliable (12%)	Reliable (11%)	Reliable (12%)	Intelligent (13%)
9	Reliable (12%)	Leader (12%)	Honest (12%)	Helpful (12%)	Intelligent (11%)	Confident (9%)	Approachable (11%)	Helpful (11%)
10	Dedicated (12%)	Caring (11%)	Trustworthy (11%)	Empathetic (12%)	Friendly (10%)	Intelligent (7%)	Dependable (11%)	Passionate (10%)

**Table 6:** Most common word choices for the prompt “How would you like your future clients to describe you?” following each word, the percentage of students using that word is in parentheses.

Year	2017	2018	2019	2020	2021	2022	2023	2024
Number of students	130	132	141	137	141	151	131	135
Rank								
1	Knowledgeable (32%)	Compassionate (38%)	Knowledgeable (38%)	Compassionate (18%)	Compassionate (40%)	Compassionate (37%)	Compassionate (36%)	Caring (39%)
2	Compassionate (30%)	Knowledgeable (34%)	Compassionate (27%)	Knowledgeable (17%)	Knowledgeable (37%)	Knowledgeable (30%)	Caring (25%)	Compassionate (33%)
3	Caring (27%)	Caring (24%)	Caring (26%)	Caring (15%)	Caring (25%)	Caring (26%)	Empathetic (22%)	Kind (31%)
4	Trustworthy (25%)	Kind (21%)	Kind (22%)	Kind (12%)	Helpful (21%)	Kind (22%)	Knowledgeable (21%)	Knowledgeable (27%)
5	Honest (18%)	Empathetic (15%)	Empathetic (21%)	Honest (12%)	Kind (20%)	Empathetic (17%)	Trustworthy (21%)	Empathetic (23%)
6	Friendly (18%)	Friendly (15%)	Helpful (17%)	Intelligent (11%)	Empathetic (16%)	Intelligent (17%)	Kind (16%)	Understanding (20%)
7	Intelligent (18%)	Helpful (15%)	Trustworthy (17%)	Empathetic (11%)	Friendly (16%)	Helpful (17%)	Helpful (15%)	Intelligent (16%)
8	Empathetic (16%)	Intelligent (15%)	Friendly (15%)	Trustworthy (9%)	Trustworthy (16%)	Understanding (17%)	Intelligent (15%)	Helpful (11%)
9	Helpful (14%)	Professional (14%)	Reliable (11%)	Hard-working (8%)	Understanding (15%)	Honest (16%)	Honest (12%)	Reliable (11%)
10	Kind (14%)	Dedicated (13%)	Intelligent (11%)	Helpful (8%)	Honest (13%)	Trustworthy (15%)	Reliable (9%)	Dedicated (10%)

**Table 7:** Most common ranked word choices for the prompt “How would you like your future clients to describe you?” when “compassionate” is combined with “empathetic” (highlighted when ranked as individual word choices).

Year	2017	2018	2019	2020	2021	2022	2023	2024
Number of students	130	132	141	137	141	151	131	135
Rank								
Combined #1	Compassionate/Empathetic (46%)	Compassionate/Empathetic (53%)	Compassionate/Empathetic (48%)	Compassionate/Empathetic (29%)	Compassionate/Empathetic (56%)	Compassionate/Empathetic (54%)	Compassionate/Empathetic (58%)	Compassionate/Empathetic (55%)
1	Knowledgeable (32%)	Compassionate (38%)	Knowledgeable (38%)	Compassionate (18%)	Compassionate (40%)	Compassionate (37%)	Compassionate (36%)	Caring (39%)
2	Compassionate (30%)	Knowledgeable (34%)	Compassionate (27%)	Knowledgeable (17%)	Knowledgeable (37%)	Knowledgeable (30%)	Caring (25%)	Compassionate (33%)
3	Caring (27%)	Caring (24%)	Caring (26%)	Caring (15%)	Caring (25%)	Caring (26%)	Empathetic (22%)	Kind (31%)
4	Trustworthy (25%)	Kind (21%)	Kind (22%)	Kind (12%)	Helpful (21%)	Kind (22%)	Knowledgeable (21%)	Knowledgeable (27%)
5	Honest (18%)	Empathetic (15%)	Empathetic (21%)	Honest (12%)	Kind (20%)	Empathetic (17%)	Trustworthy (21%)	Empathetic (23%)
6	Friendly (18%)	Friendly (15%)	Helpful (17%)	Intelligent (11%)	Empathetic (16%)	Intelligent (17%)	Kind (16%)	Understanding (20%)
7	Intelligent (18%)	Helpful (15%)	Trustworthy (17%)	Empathetic (11%)	Friendly (16%)	Helpful (17%)	Helpful (15%)	Intelligent (16%)
8	Empathetic (16%)	Intelligent (15%)	Friendly (15%)	Trustworthy (9%)	Trustworthy (16%)	Understanding (17%)	Intelligent (15%)	Helpful (11%)
9	Helpful (14%)	Professional (14%)	Reliable (11%)	Hard-working (8%)	Understanding (15%)	Honest (16%)	Honest (12%)	Reliable (11%)
10	Kind (14%)	Dedicated (13%)	Intelligent (11%)	Helpful (8%)	Honest (13%)	Trustworthy (15%)	Reliable (9%)	Dedicated (10%)

Following each word, the percentage of students using that word is in parentheses.

**Figure 2:**
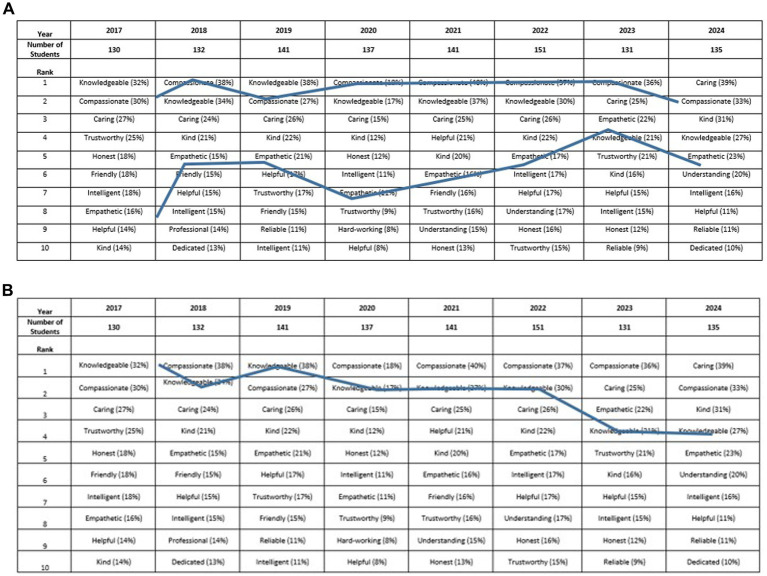
**(A)** Word trends for the prompt “How would you like your future clients to describe you?”—“Compassionate” and “Empathetic”. The percentages are the number of students that recorded this word as one of their five descriptors. “Compassionate” and “Empathetic” are not combined for this assessment. **(B)** Word trends for the prompt “How would you like your future clients to describe you?”—“Knowledgeable”. The percentages are the number of students who recorded this word as one of their five descriptors. “Compassionate” and “Empathetic” are not combined for this assessment.

Interestingly, students most often described themselves as “hard-working” from 2017 to 2019. From 2020–2024, however, there was a shift in self-description word choices to “Compassionate” and “Empathetic” as the most common descriptors recorded. When prompted about how they wanted to be described by their future veterinary colleagues, students from 2017 and 2018 most commonly used the words “Knowledgeable” or “Hard-working.” Again, there was a comparable shift in 2019 from these descriptors to recording the words “Compassionate” and/or “Empathetic” as the most common descriptor (Tables 8–10; Figure 2A). In general, students consistently wanted clients to describe them as veterinarians, as “Compassionate,” and/or “Empathetic,” and this was preserved throughout the years (Table 6). “Knowledgeable” was also a common word choice throughout the years; however, it was trending slightly downward in recent years compared with “Compassionate,” “Empathetic,” “Kind,” and or “Caring” (Figure 2B).

**Table 8:** Trends in word choices for the prompt “How would you describe yourself?” column 1 is the rank from the class of 2017, while column 4 is the rank of the same word by the class of 2024.

Prompt	“How would you describe yourself?”		
	Top 10-word choices		
Rank 2017		Rank trend	Rank 2024
1	Hard-working	Top choice 2017–2020 and 2024; Remained within top 3	1
2	Determined	Dropped and then slowly increased	3
3	Responsible	Fell off list after 2017	Not in Top 10
4	Dedicated	Increased initially then decreased; fell out of top 10 after 2021	Not in Top 10
5	Reliable	First 3 years on list; fell off list in after 2019	Not in Top 10
6	Helpful	Increased initially then decreased; fell out of top 10 after 2019	Not in Top 10
7	Friendly	First 2 years on list; fell off list in after 2019	Not in Top 10
8	Motivated	First 2 years on list; fell off list in after 2018	Not in Top 10
9	Dependable	Only one year on list; fell off list in after 2017	Not in Top 10
10	Professional	Only one year on list; fell off list in after 2017	Not in Top 10
Not ranked	Kind	Entered list 2018; increasing rank	2
Not ranked	Caring	Entered list 2018; increasing rank	4
Not ranked	Compassionate	Entered list 2018; increasing rank	5
Not ranked	Empathetic	Entered list 2020; increasing rank	6
Not ranked	Passionate	Entered list 2019; increasing rank	7
Not ranked	Loyal	Only on 2024 list	8
Not ranked	Driven	Entered list 2020; stable rank	9
Not ranked	Organized	Only on 2024 list	10

Highlighted are the word choices of the top 10 word choices from the class of 2024. Column 3 describes the trend over the word used in column 2. “Compassionate” and “empathetic” are not combined for this assessment.

**Table 9:** Trends in word choices for the prompt “How would you like your future colleagues to describe you?” column 1 is the rank from the class of 2017, while column 4 is the rank of the same word by the class of 2024.

Prompt	“How would you like your future colleagues to describe you?”		
	Top 10-word choices		
Rank 2017		Rank trend	Rank 2024
1	Knowledgeable	Top one or two choices 2017–2021; declined after	7
2	Hard-working	Top one, two choice 2017–2020; Second choice 2022–2024	2
3	Compassionate	Top four 2017–2020; top choice 2021–2024	1
4	Intelligent	Trended down except for 2023	8
5	Caring	Relatively stable rank	5
6	Passionate	Variable rank	10
7	Trustworthy	Trended down; off the list after 2019	Not in Top 10
8	Helpful	Variable, however, relatively stable in 5 to 10 ranks	9
9	Reliable	Variable however relatively stable at lower rank	4
10	Dedicated	Variable however trending upward	3
Not ranked	Kind	Entered list 2019, increasing rank	6

Highlighted are the word choices of the top 10 word choices from the class of 2024. Column 3 describes the trend over the word used in column 2. “Compassionate” and “Empathetic” are not combined for this assessment.

**Table 10:** Trends in word choices for the prompt “How would you like your future clients to describe you?” column 1 is the rank from the class of 2017, while column 4 is the rank of the same word by the class of 2024.

Prompt	“How would you like your future clients to describe you?”		
	Top 10-word choices		
Rank 2017		Rank trend	Rank 2024
1	Knowledgeable	Ranked 1 or 2 through 2022; decreased slightly after	4
2	Compassionate	Ranked 1 or 2 through 2019; Ranked 12020–2023	2
3	Caring	Generally ranked 3; increased 2023 and 2024	1
4	Trustworthy	Variable on the list; trended down except for 2023	Not ranked
5	Honest	Variable on the list; trended down	Not ranked
6	Friendly	Variable on the list; trended down through 2021; not on list after	Not ranked
7	Intelligent	Trended down; then off list; back in 2023 and 2024	7
8	Empathetic	Trending upward	5
9	Helpful	Variable, however, relatively stable in 5 to 10 ranks	8
10	Kind	Trending upward	3
Not ranked	Understanding	Entered list 2021; slight increase	6
Not ranked	Reliable	Entered list 2019; variable position 5 to 10 rank	9
Not ranked	Dedicated	Only on list in 2024	10

Highlighted are the word choices of the top 10 word choices from the class of 2024. Column 3 describes the trend over the word used in column 2. “Compassionate” and “Empathetic” are not combined for this assessment.

## Discussion

In this assignment, first-year students self-reflected on their current and future selves in the process of conceptualizing a future “professional self.” We often find student responses such as these useful in developing various follow-up educational activities and discussions. We chose to collect these data from first-year veterinary students as the concept of professional “identity” was a subsequent class discussion (the next class week following completion of the assignment), and the grouped student responses were used to frame that subsequent class discussion.

In summarizing these responses, we decided to present this information with descriptive statistics given the self-reflective nature of the assignment and as this was a unique cohort from only one institution from one country. Clearly, as important factors such as environment and culture may influence any student’s response, each institution will likely need to collect similar data to contextualize the results for that institution’s curriculum development. Our process offers one potential mechanism for the collection of this information and a base of comparison.

As the students were free to choose any potential word choice to answer these self-reflective questions, the words they recorded are inherently interesting to us as educators in our individual institution and contribute to our understanding of students’ self-view both now and in the future. Overall, students recorded a range of descriptors compared with a finite or uniform group of self-descriptive word choices. As individuals normally vary in self-reflective word choices, this diversity in word choices was not unexpected (15). It is notable, however, that relatively fewer unique word choices were recorded when asked how the students wanted to be described by future clients. This prompts several questions: Could these results indicate that students generally have more limited and/or universal views of their identity goals relative to future client expectations as compared to self or future colleagues? Or is this an inherent bias because of the question sequencing, given that the question relative to future client views was the third of the series and, therefore, the students defaulted to word choices that they had already recorded in prior questions (i.e., an availability bias)? Or do students believe that clients have inherently standardized expectations for them as veterinarians, and therefore, the students implicitly understand these client expectations and just articulate them? As we did not investigate further with the students in this assignment about why they chose the words that they did, we could not answer these questions definitively with the data available from this exercise. These questions, however, do provide opportunities for further student discussion during future curricular activities.

Fundamentally, individuals should be, but also may not be, the most appropriate assessors of their own skills and attributes when asked to perform various self-assessments (6, 10, 13, 14, 18, 28, 40, 45, 46, 51, 53, 57, 58, 65). When the performance criteria are objective and measurable, individuals are usually more accurate in rating their own skills relative to these benchmarks. When the assessments are less quantifiable and/or theoretical, such as “competence,” individuals are less accurate in their self-assessments relative to an external objective standard. For this study, we were not intending to determine if these students are, in fact, what they say they are or will be. More so, we were interested in what they were thinking about themselves now and in the future as veterinary professionals.

Relative to self and future colleague descriptors, there was an interesting shift from the initial student cohorts (e.g., 2017 and 2018) from words “Hard-working” and “Knowledgeable” to later cohorts’ words of “Compassionate,” “Empathetic,” and “Caring.” As each student cohort was relatively early in the veterinary curriculum and had not been explicitly exposed to concepts of “Compassion” and “Empathy” within our curriculum at this point, it seems reasonable to conclude that these frames of reference developed prior to entering the veterinary curriculum or potentially within the first weeks of their educational activities. As professional identity is a dynamic process, it is likely that some influence on student word choices was the result of previous veterinary-related experiences prior to entering the curriculum.

Within the limitations inherent in this unique dataset, there are clear themes that emerged from these results that prompt educational consideration. As might be expected, students chose descriptors such as “Hard-working,” “Knowledgeable,” “Compassionate,” and “Empathetic” as both current and future descriptors. In a medical context, it seems intuitively reasonable, at least for clinically focused individuals, to desire to be described as “Caring” for others.

The words “Compassionate” and “Empathetic” were subsequently combined for an independent analysis, given that individuals frequently use these words interchangeably and without a common agreement on the difference in meaning. While “Compassionate” and “Empathetic” were often chosen as a descriptor, when combined, “Compassionate” and “Empathetic” were the top-ranked words recorded by 46 to 58% of students (depending on the year) when answering the question about how they want to be described by future clients. The fall 2020 cohort was the one outliner with just under 30% of students recording one or the other of these terms. Data from other studies of veterinary and client surveys have identified the influence of similar word choices on both professional stereotypes and on client expectations for veterinary behavior (3, 8, 47, 67). In one study (67), on evaluating reasons for potential litigation against veterinarians, clients noted “Compassion” and “Empathy,” or lack thereof, as important in their decision-making process to pursue litigation. This fact emphasizes the importance of projecting a professional “identity,” such as “Compassionate,” and most importantly, to be intentional in doing so, given the impact of these professional identities on self and others.

It deserves equal consideration that collectively 40% of students did not record either “Compassionate” or “Empathetic” as a current or future descriptor. Does this suggest that being or viewed as being “Compassionate” and/or “Empathetic” is not an identity goal of some students? Or did these students assume these descriptors were inherent as a professional expectation and choose other words instead? Do students with certain future career tracks, for example, not view these descriptors as necessary professional “identities”? While we did not independently correlate word choice with career choice in this analysis, at our institution, first-year veterinary students’ future career interests trended upward in the companion animal and or companion animal mixed focus during the study period. This prompts future analysis, for example, related to the influence of choice of career focus and the necessity to be viewed as “Compassionate” or “Empathetic.” Additionally, are we choosing, either explicitly or implicitly, for certain professional “identities through our admission processes? Are individuals with these behavioral characteristics inherently choosing the veterinary profession because of preconceived professional stereotypes prior to veterinary college? These questions may be answerable in the future through the analysis of our admission evaluations and other selection criteria for accepting students into the veterinary curriculum.

Our results do potentially create a dilemma for educators in developing curriculum to enhance compassion and empathy as a competency (9, 23, 29–33, 35–38, 41, 50, 52, 54–56, 59, 60). While many students would likely engage in developing this type of skill development, should all students, even if not their individual professional priority, be exposed to educational activities such as compassion training? Similarly, whether developing compassion is or should be an educational outcome or competency of the veterinary curriculum? Our bias, certainly for clinically focused individuals, is that the development of “compassion” as a foundational clinical skill is imperative for medical professionals and contributes positively to future success in direct patient/client interactions, as well as the global reputation of the veterinary profession.

Finally, there is another important question for consideration: Does compassion develop organically during the veterinary educational process? Data from some human medical settings suggests “no” or not necessarily (41). In some environments, especially as students develop more medical knowledge, their development of “Compassion” may, in fact, decrease (41). This dynamic may be a contributing factor to compassion fatigue and other negative perceptions of the profession.

If the development of compassion is a competency outcome goal, then there is evidence that incorporation of specific exercises to develop compassion as a skill can be helpful (9, 23, 29–33, 35–38, 41, 50, 52, 54–56, 59, 60). As an example, training in a compassion meditation has been shown to result in actual alterations in brain structure as an outcome (32, 33). In our curriculum, we have both explicit and implicit opportunities for students to develop knowledge and skills in being “compassionate.” As an example, students are explicitly asked in our curriculum to explore the meaning of the words “Compassion” and “Empathy” in both the first year “Careers” class and in the third year “Communication” class. These are core curriculum classes that are components of a series of courses primarily to develop the “non-technical” skills of veterinary students. In the “Communication” course, students have four interactions with simulated clients, and these clients’ evaluation rubric includes an assessment of whether the student was “Compassionate.” Following the simulated client interaction, the students are provided direct feedback as to whether the client felt that they were, in fact, “Compassionate” toward them and their animal. Additionally, students in the first year become Fear-Free certified as implicitly fostering a compassionate viewpoint of animal handling (16). While not always explicit, role modeling by faculty and staff in how to demonstrate compassion is likely the most important influence on the students’ future behaviors in this competency. This facet of implicit education is dependent on the individual faculty and staff member and is, therefore, an uncontrolled variable. We should, however, ask the important question: Are we role modeling compassionate behaviors in both the formal and “hidden” curriculum? (3, 25, 42, 61). Based on the results of our analysis and the common themes that emerged, it seems reasonable that we should be explicit in teaching “Compassion” as a clinical skill in clinically-focused individuals and to consistently role model that behavior in teaching laboratories, preceptors, and other educational activities external to the veterinary college and in clinical practices that we use for student education.

In conclusion, as veterinary educators and as future veterinary colleagues as well as potential clients, it seems critically important that we recognize and understand both the preserved as well as the changing perspectives of students relative to a range of aspects of the veterinary career to maintain contemporary in our curriculum and professional delivery, and equally to understand what is important to the modern veterinary student. Hopefully, the characteristics and outcome behaviors we instill in our students align with their future “identity” for our profession as these students enter the workforce as veterinary professionals.

## Data Availability

The raw data supporting the conclusions of this article will be made available by the authors, without undue reservation.

## Footnotes

^1^ https://www.qualtrics.com/
